# Inflammation is correlated with severity and outcome of cerebral venous thrombosis

**DOI:** 10.1186/s12974-018-1369-0

**Published:** 2018-11-26

**Authors:** Liyan Wang, Jiangang Duan, Tingting Bian, Ran Meng, Longfei Wu, Zhen Zhang, Xuxiang Zhang, Chunxiu Wang, Xunming Ji

**Affiliations:** 1 Neurosurgery Department of Xuanwu Hospital Capital Medical University, Changchun st 45, Xicheng District, Beijing, China; 2 Emergency Department of Xuanwu Hospital Capital Medical University, Changchun street 45, Xicheng District, Beijing, China; 3 Ophtalmology Department of Xuanwu Hospital Capital Medical University, Changchun street 45, Xicheng District, Beijing, China; 4 Evidence-based Medicine Department of Xuanwu Hospital Capital Medical University, Changchun street 45, Xicheng District, Beijing, China

**Keywords:** Cerebral venous thrombosis, Inflammation, Stage, Severity, Outcome

## Abstract

**Background:**

Few studies have suggested a relationship between inflammation and cerebral venous thrombosis (CVT). This retrospective study aimed to explore the changes in inflammation in different CVT stages and the correlation between inflammation and severity and outcome of CVT.

**Methods:**

In total, 95 suitable patients with CVT and 41 controls were compared. Patients with CVT were divided into three groups. The inflammatory factors studied included hypersensitive C-reactive protein (Hs-CRP), interleukin-6 (IL-6), and neutrophil-to-lymphocyte ratio (NLR) in the peripheral blood and immunoglobulin A (IgA), immunoglobulin M (IgM), and immunoglobulin G (IgG) in the cerebrospinal fluid (CSF). The severity of CVT was evaluated with the modified Rankin Scale (mRS), the National Institutes of Health Stroke Scale (NIHSS), fundus condition, intracranial pressure (ICP), and complications on admission. The short-term outcome was evaluated with the mRS at discharge.

**Results:**

The following results were obtained: (1) Inflammatory factor levels in patients with CVT were higher than those in the controls. (2) Inflammatory factor levels in the acute and subacute stages were significantly higher than those in the chronic stage (all *P* < 0.05). (3) Serum NLR and CSF IgM levels were positively related to baseline degree of disability (odds ratio [OR], 1.279, 95% confidence interval [CI] 1.009–1.621, *P* = 0.042; OR 1.402, 95% CI 1.036–1.896, *P* = 0.028). The Hs-CRP level was positively correlated with the baseline occurrence of seizure (OR 1.040, 95% CI 1.001–1.080, *P* = 0.043). The baseline serum NLR (*r* = 0.244, *P* = 0.017), CSF IgA (*r* = 0.615, *P* < 0.001), CSF IgM (*r* = 0.752, *P* < 0.001), and CSF IgG (*r* = 0.248, *P* = 0.015) levels were positively associated with NIHSS. (4) The baseline NLR was significantly associated with high risk of poor outcome at discharge (OR 1.339, 95% CI 1.097–1.784, *P* = 0.007). Moreover, the ROC showed that NLR ≥ 4.205 could better predict the poor outcome at discharge. The data were analyzed using SPSS.

**Conclusions:**

Inflammation may develop after CVT and gradually decrease during the course. Inflammation was significantly correlated with severity on admission and short-term poor outcome at discharge in CVT.

## Background

Unlike artery ischemic stroke, cerebral venous thrombosis (CVT) is an uncommon type of stroke mainly developing in the young and middle-aged population, especially women [1]. Currently, the incidence of CVT is higher than 1.3–1.6/100,000 in Western countries and probably even higher in Asia and the Middle East [2]. The risk factors could be divided into inflammatory factors including infection or nonspecific inflammation of the head and face or other sites and non-inflammatory factors such as hypercoagulability, blood stasis, vascular wall injury, and intracranial hypotension [3]. The current main medical treatments of CVT include anticoagulation, etiological treatment, and symptomatic therapy [1]. Over the past decades, the mortality has declined because of improved diagnostics and early treatment with anticoagulation. However, many patients recovering without evident physical disability may have residual chronic symptoms consisting of headache or neuropsychological problems and the like [2]. Hence, we want to explore some undefined therapeutic targets and potential treatments to improve the prognosis of CVT.

Anecdotal evidences have showed the extensive crosstalk between inflammatory cytokines and coagulation factors, indicating that inflammation and coagulation are correlative [4]. Numerous studies have found that inflammation plays vital roles in many thromboembolic diseases such as acute ischemic stroke (AIS), deep venous thrombosis (DVT), and pulmonary embolism (PE) [5–7]. Furthermore, inflammation can be the cause of CVT [8]. However, the relationship between inflammation and CVT in patients without definite inflammatory diseases remains unknown.

Recently, a number of studies have focused on determining the appropriate inflammatory factors to predict the outcome of patients with various thromboembolic diseases. As clinically easily available inflammatory factors, the increased hypersensitive C-reactive protein (Hs-CRP), interleukin-6 (IL-6) levels, and neutrophil-to-lymphocyte ratio (NLR) in the peripheral blood have been confirmed to be correlated with poor outcome in AIS, DVT, and PE [6, 9, 10]. CRP is a blood biomarker produced by the liver representing acute-phase systemic inflammation. Hs-CRP can accurately detect low-grade inflammation and is widely used in clinical practice particularly in cerebrovascular and cardiovascular diseases [11, 12]. IL-6 is a significant inflammatory factor produced by various cells including endothelial cells. Previous animal and clinical experiments have revealed that IL-6 participates in inflammation during coagulation partly through provoking stimulation of acute-phase reactants or chemoattractants [13, 14]. Moreover, IL-6 was correlated with venous thrombosis [15]. Recently, the NLR, which was associated with poor outcome in various vascular diseases of the heart and brain, also turns out to be a relatively reliable indicator of ongoing destructive inflammation [16–18]. Moreover, NLR was believed to be more stable and valuable than single changes in neutrophil or lymphocyte [19].

CVT is a type of cerebrovascular disease. Hence, evaluating inflammation of the brain is necessary. Cerebrospinal fluid (CSF) is produced in the brain, circulates throughout the central nervous system (CNS), and is located between the brain and skull [20]. The CSF can reflect the biochemical changes occurring in the brain because of its direct contact with the extracellular space of the brain [21]. It is well known that the significant elevation of CSF immunoglobulin (Ig) could indicate inflammatory reaction in the CNS [22].

To investigate the changes in inflammation in different stages of CVT and the correlation between inflammation and severity and outcome of CVT, we conducted this study and selected the above inflammatory biomarkers as research indicators. Hopefully, we could provide evidence for potential anti-inflammatory treatment on patients with CVT without definite inflammatory diseases to improve the outcome.

## Methods

### Study design

We retrospectively identified 95 newly diagnosed patients with CVT and 41 age- and sex-matched individuals for the control group admitted to our hospital from March 2015 to March 2018. Inclusion criteria for our study were newly diagnosed CVT on magnetic resonance imaging (MRI), magnetic resonance venography (MRV), computed tomographic venography (CTV), or conventional digital subtraction angiography (DSA) [1, 23]. The age and sex were unlimited. The individuals in the control group were those who were admitted to our hospital with suspicious CVT but diagnosed with other non-inflammatory diseases at discharge. Patients and control individuals were excluded if they have the following conditions [9]: AIS, acute myocardial infarction, renal or hepatic failure, malignancy, DVT and PE; definite immune diseases such as connective tissue diseases, rheumatic diseases; definite acute or chronic infection; use of anti-inflammatory medication within 4 weeks prior to blood collection; and refusal of blood sampling and lumbar puncture. All patients received the formal anticoagulation treatment. All protocols were approved by the Institutional Review Board.

### Data collection

The following data of included patients with CVT were obtained at baseline in the study: demographics; risk factors for CVT; dates of onset of symptoms; symptoms and signs from onset to diagnosis; the features of imaging; the National Institutes of Health Stroke Score (NIHSS); modified Rankin Scale (mRS); the fundus condition; the intracranial pressure (ICP) [cerebrospinal fluid (CSF) opening pressure obtained by lumbar puncture in recumbent position)]; CVT-related complications, including intracranial hemorrhage, cerebral venous infarction and seizure; levels of D-dimer, hypersensitive C-reactive protein (Hs-CRP), interleukin-6 (IL-6), and the neutrophil-to-lymphocyte ratio (NLR) in peripheral blood and immunoglobulin A (IgA), immunoglobulin M (IgM), and immunoglobulin G (IgG) in CSF measured within 24 h after admission. The mRS score at discharge was also collected. Additionally, the mRS score, the fundus condition, and ICP at 3 to 12 months after the discharge were also documented. The data on age, sex, and six abovementioned inflammatory factors were also collected in the control group.

### Clinical assessment

The severity of CVT on admission was assessed by NIHSS, mRS, presence of papilledema, intracranial hypertension (IH), cerebral venous infarction, intracranial hemorrhage, and seizure. The mRS ≤ 1 was defined as good condition and mRS > 1 was defined as poor condition [24]. Fundus photography detected the presence of papilledema. An ICP of more than 200 mmH2O was defined as intracranial hypertension.

The short-term outcome of CVT was evaluated with mRS at discharge [25]. The mRS ≤ 1 was defined as good outcome and mRS > 1was defined as poor outcome. The long-term outcome of CVT was assessed by mRS, presence of papilledema, and IH at 3 to 12 months after discharge.

### Statistical analysis

All statistical analyses were performed by SPSS. Quantitative variables with a normal distribution were specified as mean ± standard deviation, and those with an abnormal distribution were expressed as median with interquartile range (IQR). Categorical variables were specified with number and percentage (%) values. Student’s *t* test or Mann–Whitney test was used for continuous data, while *χ*^2^ or Fisher’s exact test was used for categorical data. The independent variables associated with the severity and outcome of CVT were analyzed by logistic regression analysis. Possible confounding factors were tested in a univariable regression analysis, and then, confounders with a *P* value < 0.05 were tested in a multivariable logistic regression analysis. The correlation between baseline NIHSS and inflammatory factors were assessed by Spearman’s correlation coefficients. The receiver operating characteristic (ROC) curve was used to demonstrate the sensitivity and specificity of significant variables and the optimal cutoff values for predicting the outcome. A difference between the groups was considered significant if *P* < 0.05.

## Results

### Patients

A total of 95 patients with CVT (mean age, 38.93 ± 13.53 years, and 60.0% were female) and 41 controls (mean age, 40.90 ± 16.70 years, and 63.41% were female) were included in the study. In patients with CVT, 23.16% were in the acute stage, 32.63% in the subacute stage, and 44.21% in the chronic stage; 41.05% of patients were in poor condition (mRS > 1), 68.35% had IH, 60.56% had papilledema, 38.95% had cerebral venous infarction, 25.26% had seizure, and 11.58% had intracerebral hemorrhage. Of the patients, 30.53% had incomplete follow-up information and were removed from the follow-up data analysis.

### Inflammation between patients with CVT and controls

Compared with the controls, the patients with CVT had higher Hs-CRP (3.3 [0.7, 11.73] vs 0.5[0.26, 1.6],*P* < 0.001), IL-6 (9.49 [5.76, 14.94] vs 4.32 [3.01, 5.02], *P* = 0.003), NLR (2.84 [1.86, 3.73] vs 2.05 [1.77, 2.59], *P* < 0.001), CSF IgA (0.4 [0.24, 0.81] vs 0.2 [0.13, 0.23], *P* < 0.001), CSF IgM (1.3 [0.5, 2.6] vs 0.04 [0.02, 0.08], *P* < 0.001), and CSF IgG (3.48 [2.22, 5.56] vs 2.54 [1.98, 2.88], *P* = 0.001) (Table 1).

**Table 1:** The clinical features of the two groups

	CVT patients *n* = 95	Control individuals *n* = 41	*P* value
Age, years	38.93 ± 13.53	40.90 ± 16.70	0.469
Gender (female, %)	60	63.41	0.708
Hs-CRP	3.3 (0.7, 11.73)	0.5 (0.26, 1.6)	< 0.001
IL-6	9.49 (5.76, 14.94)	4.32 (3.01, 5.02)	0.003
NLR	2.84 (1.86, 3.73)	2.05 (1.77, 2.59)	< 0.001
CSF IgA	0.4 (0.24, 0.81)	0.2 (0.13, 0.23)	< 0.001
CSF IgM	1.3 (0.5, 2.6)	0.04 (0.02, 0.08)	< 0.001
CSF IgG	3.48 (2.22, 5.56)	2.54 (1.98, 2.88)	0.001

*CVT* cerebral venous thrombosis, *Hs-CRP* hypersensitive C-reactive protein, *Il-6* interleukin-6, *NLR* the neutrophil to lymphocyte ratio, *IgA* immunoglobulin A, *IgM* immunoglobulin M, *IgG* immunoglobulin G, *CSF* cerebrospinal fluid

### Inflammation and stages of CVT

**Fig. 1:**
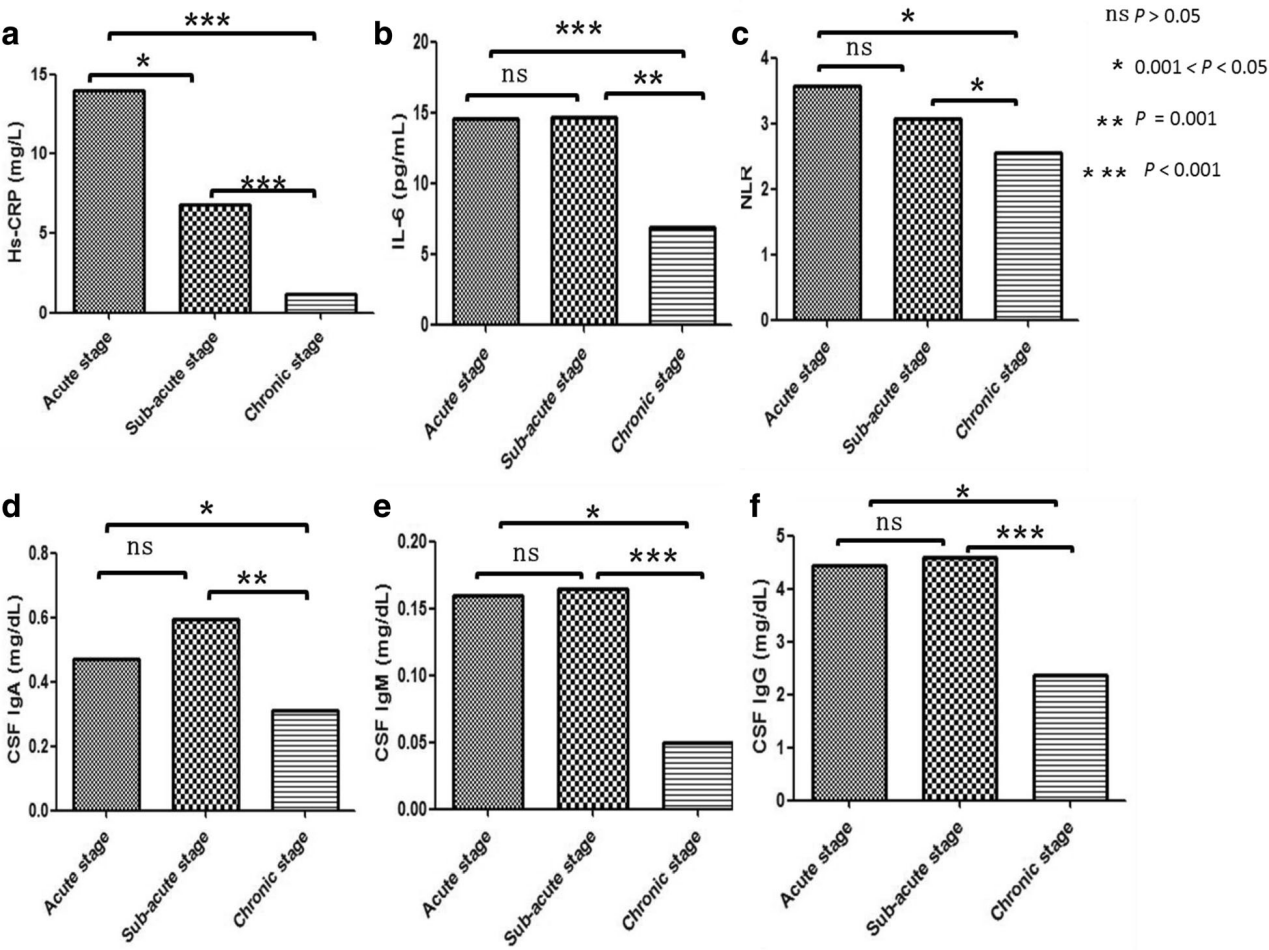
**a** The Hs-CRP level in the acute and sub-acute stages were higher than that in the chronic stage, and its level in the acute stage was higher than that in the subacute stage. **b** The IL-6 level in the acute and sub-acute stages were significantly higher than that in the chronic stage. **c** The NLR level in the acute and sub-acute stages were higher than that in the chronic stage. **d** The CSF IgA level in the acute and sub-acute stages were higher than that in the chronic stage. **e** The CSF IgM level in the acute and sub-acute stages were higher than that in the chronic stage. **f** The CSF IgG level in the acute and sub-acute stages were higher than that in the chronic stage

The levels of most inflammatory factors between acute and subacute stages have no difference (*P* > 0.05). The Hs-CRP level in the acute stage was higher than that in the subacute stage. The levels of all inflammatory factors in the acute and sub-acute stages were higher compared with those in the chronic stage (Hs-CRP, 14.03 [4.93, 28.42] vs 1.16 [0.26, 2.95], *P* < 0.001, and 6.83 [1.51, 14.15] vs 1.16 [0.26, 2.95], *P* < 0.001; NLR, 3.58 [2.63, 5.24] vs 2.56 [1.66, 3.15], *P* = 0.003, and 3.08 [2.15, 4.65] vs 2.56 [1.66, 3.15], *P* = 0.043; IL-6, 14.58 [8.93, 14.94] vs 6.82 [4.81, 8.01], *P* < 0.001, and 14.63 [6.73, 16.14] vs 6.82 [4.81, 8.01], *P* = 0.001; CSF IgA, 0.47 [0.25, 0.91] vs 0.31 [0.15, 0.48], *P* = 0.046, and 0.60 [0.29, 1.01] vs 0.31 [0.15, 0.48], *P* = 0.001; CSF IgM, 0.16 [0.03, 0.53] vs 0.05 [0.03, 0.09], *P* = 0.026, and 0.17 [0.10, 0.24] vs 0.05 [0.03, 0.09], *P* < 0.001; CSF IgG, 4.45 [2.50, 7.61] vs 2.36 [1.61, 3.19], *P* = 0.002, and 4.60 [3.05, 5.85] vs 2.36 [1.61, 3.19], *P* < 0.001) (Fig. 1).

### Inflammation and the severity of CVT

**Fig. 2:**
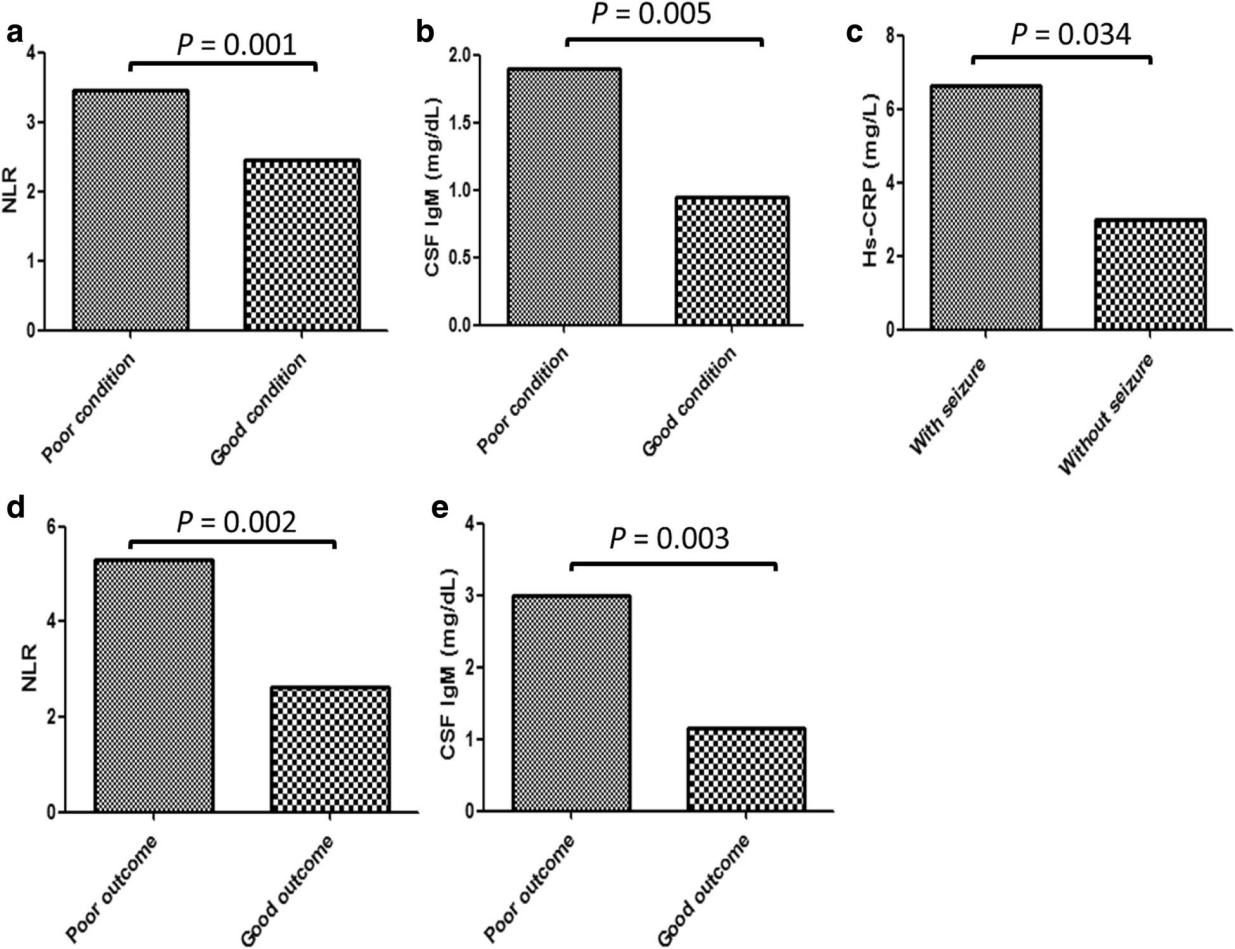
**a**, **b** The difference in NLR and CSF IgM level between patients with good condition and those with poor condition. **c** The difference in Hs-CRP level between patients with seizure and those without seizure. **d**, **e** The difference in NLR and CSF IgM level between patients with good outcome and those with poor outcome

Spearman’s correlation analysis showed that the level of serum NLR (*r* = 0.244, *p* = 0.017), CSF IgA (*r* = 0.615, *p* < 0.001), CSF IgM (*r* = 0.752, *p* < 0.001), and CSF IgG (*r* = 0.248, *p* = 0.015) were positively associated with baseline NIHSS. We conducted a logistic regression analysis on the correlation between inflammation and degree of disability defined by mRS, IH, papilledema, cerebral venous infarction, seizure, and intracranial hemorrhage. The independent variables were the levels of six inflammatory factors, age, and sex. The results were as follows: the serum NLR [adjusted odds ratio (OR), 1.279; 95% confidence interval (*CI*) 1.009–1.621, *P* = 0.042], and CSF IgM (adjusted OR, 1.402; 95% *CI* 1.036–1.896, *P* = 0.028) level were positively associated with baseline degree of disability. The Hs-CRP level was positively associated with the development of seizure at baseline (adjusted OR 1.040; 95% *CI* 1.001–1.080, *P* = 0.043). All 95 patients were grouped respectively based on mRS and seizure on admission: patients with good and poor condition and those with and without seizure. The NLR and CSF IgM level were significantly higher in patients with poor condition compared with those with good condition (3.45 [2.31, 5.29] vs 2.45 [1.72, 3.14], *P* = 0.001; and 1.90 [0.60, 3.00] vs 0.95 [0.40, 2.02], *P* = 0.005) (Fig. 2a, b). The Hs-CRP level was higher in patients with seizure than that in patients without seizure (6.64 [0.94, 35.72] vs 3.12 [0.54, 9.22], *P* = 0.034) (Fig. 2c).

Although the univariate logistics analysis revealed the correlation between baseline cerebral venous infarction and serum Hs-CRP (OR 1.053; 95% *CI* 1.016–1.092, *P* = 0.005), CSF IgM (OR 1.377, 95% *CI* 1.049–1.809, *P* = 0.021), and CSF IgG levels (OR 1.291, 95% *CI* 1.071–1.556, *P* = 0.007), the multivariate logistics analysis found no relationship between them (all *P* > 0.05). However, there was no significant relationship between inflammatory factors and baseline IH and papilledema (all *P* > 0.05).

### Inflammation and outcome of CVT

All 95 patients were divided into two groups based on mRS at discharge: patients with good outcome and poor outcome. Baseline characteristics of the patients in two groups are shown in Table 2. There was no statistical difference between the two groups with regard to age, sex, stages, duration of hospital stay, and serum Hs-CRP, IL-6, CSF IgA, and CSF IgG levels (all *P* > 0.05). Compared with patients with good outcome, patients with poor outcome had higher NIHSS score (median 2.00 [1.00, 7.50] vs 0.00 [0.00, 1.00], *p* < 0.001), NLR (median 5.29 [3.00, 7.26] vs 2.62 [1.79, 3.44], *p* = 0.002), and CSF IgM level (median 3.00 [1.65, 6.22] vs 1.15 [0.48, 2.33], *p* = 0.003) (Fig. 2d, e).

**Table 2:** Data of patients with good and poor condition on admission

	Poor condition *n* = 39	Good condition *n* = 56	*P* value
Female (%)	27/39 (69.23%)	30/56 (53.57%)	0.142
Male (%)	12/39 (30.77%)	26/56 (46.43%)	0.142
Age, years	37.62 ± 11.67	39.84 ± 14.72	0.434
Serum Hs-CRP (mg/L)	4.88 (0.86, 20.42)	3.06 (0.65, 6.74)	0.194
Serum NLR	3.45 (2.31, 5.29)	2.45 (1.72, 3.14)	0.001
Serum IL-6 (pg/ml)	11.87 (6.50, 14.94)	8.01 (4.98, 14.94)	0.451
CSF IgA (mg/dl)	0.46 (0.21, 0.90)	0.38 (0.38, 0.74)	0.617
CSF IgM (mg/dl)	1.90 (0.60, 3.00)	0.95 (0.40, 2.02)	0.005
CSF IgG (mg/dl)	4.16 (1.87, 6.80)	3.22 (2.29, 4.96)	0.423

*CVT* cerebral venous thrombosis, *Hs-CRP* hypersensitive C-reactive protein, *Il-6* interleukin-6, *NLR* the neutrophil to lymphocyte ratio, *IgA* immunoglobulin A, *IgM* immunoglobulin M, *IgG* immunoglobulin G, *CSF* cerebrospinal fluid

Logistic regression was used to study the value of inflammatory factors in predicting the outcomes at discharge. The independent variables were the baseline levels of six inflammatory factors, age, sex, and NIHSS score. The multivariate logistics analysis found that the baseline NLR was significantly associated with high risk of disability at discharge (adjusted OR, 1.339; 95% *CI* 1.097–1.784, *P* = 0.007).

**Fig. 3:**
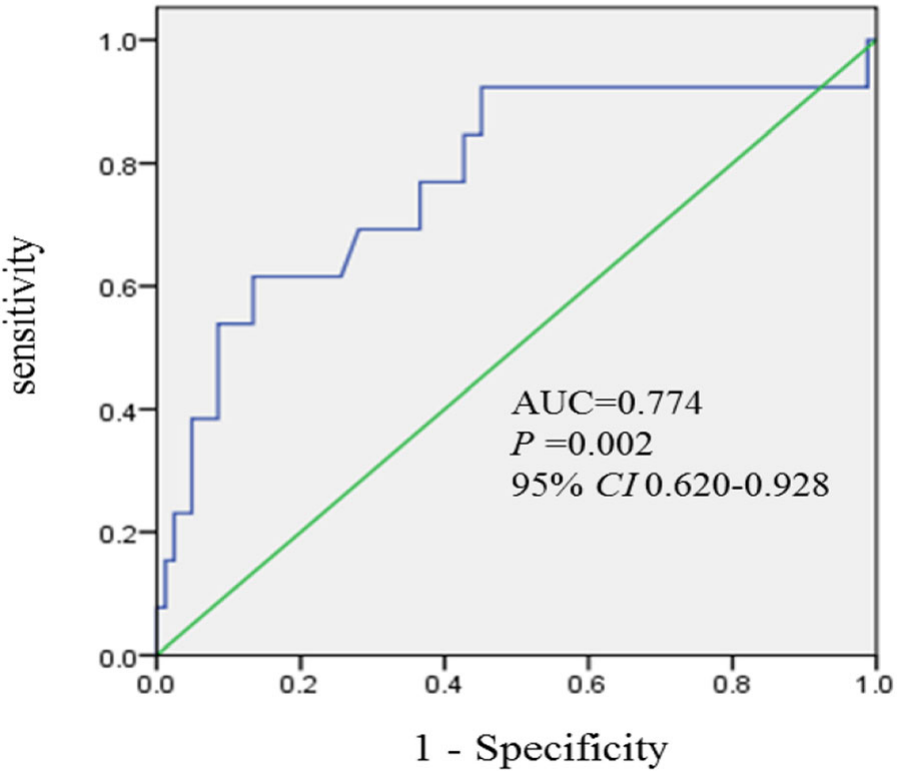
Receiver operating characteristic (ROC) curve for serum NLR on predicting short-term outcome of non-inflammatory CVT. CVT, cerebral venous thrombosis; AUC, area under the curve; CI, confidence interval; NLR, neutrophil-to-lymphocyte ratio

The ROC curve demonstrated the predicting power of baseline serum NLR on the short-term outcome with an area under the curve value of 0.774 (*P* = 0.002, 95% *CI* 0.620–0.928) (Fig. 3). The optimal cutoff is 4.205 (positive predictive value 61.5%, negative predictive value 86.6%, + likelihood ratio [LR] 1.60, − LR 0.15). To further estimate the significance of baseline NLR on predicting the outcome of CVT, patients were divided into two groups according to the cutoff value of NLR (NLR < 4.205 and ≥ 4.025), and the details are shown in Table 3. The results revealed that the group with NLR ≥ 4.205 had higher NIHSS scores (median 2.00 [0.00, 7.00] vs 0.00 [0.00, 1.00], *P* < 0.001) and poor outcome (42.11% vs 6.58%, *P* < 0.001).

**Table 3:** Data of patients classified according to NLR subgroup

	NLR < 4.205 *n* = 76	NLR ≥ 4.205 *n* = 19	*P* value
Male (%)	28/76 (36.84)	10/19 (52.63)	0.209
Female (%)	48/76 (63.16)	9/19 (47.37)	0.209
Age, years(mean ± SD)	38.01 ± 13.23	42.58 ± 14.45	0.190
NIHSS score on admission (median, IQR)	0.00 (0.00, 1.00)	2.00 (0.00, 7.00)	< 0.001
Seizure (%)	17/76 (22.37)	7/19 (36.84)	0.194
Infarction (%)	28/76 (36.84)	9/19 (47.37)	0.400
Intracerebral hemorrhage (%)	7/76 (9.21)	4/19 (21.05)	0.149
Papilledema (%)	33/76 (43.42)	9/19 (47.37)	0.757
Intracranial hypertension (%)	53/76 (69.74)	13/19 (68.42)	0.911
Poor outcome (%)	5/76 (6.58)	8/19 (42.11)	< 0.001

*NIHSS* the National Institutes of Health Stroke Score, *IQR* interquartile range

During the follow-up, 69.47% (66/95) of patients completed the telephone follow-up, and 53.68% (51/95) underwent lumbar puncture and fundus examination. The IL-6 level (OR, 1.063; 95% *CI* 1.008–1.122, *P* = 0.025) and NIHSS score (OR, 1.264; 95% *CI* 1.001–1.596, *P* = 0.049) were associated with high risk of poor outcome in the univariable model. However, the multivariate logistics analysis found no significant relationship (all *P* > 0.05). Moreover, the levels of these inflammatory factors were not correlated with the prognosis of non-inflammatory CVT including the development of IH and papilledema (all *P* > 0.05).

## Discussion

It is well known that inflammation plays vital roles in artery stroke, which not only participates in the development of stroke but also plays a continuing role during the various stages of stroke and influences the outcome [26–29]. Similarly, inflammation could act as the cause of CVT, which has been demonstrated by numerous previous studies [8]. However, whether the inflammation develops after CVT and is associated with its severity and outcome is not yet presently defined.

As clinically easily available inflammatory biomarkers, Hs-CRP, IL-6, and NLR in the peripheral blood and Ig (A, M, G), in the CSF were used in our study to represent the inflammatory response. According to our results, the levels of abovementioned six inflammatory factors were higher in patients with CVT compared with those in the controls. Furthermore, these inflammatory factors including Hs-CRP, IL-6, and NLR in the peripheral blood and IgA, IgM, and IgG in the CSF increased significantly during the acute and subacute stages and decreased during the chronic stage. It indicated that inflammation may develop soon after CVT and gradually decrease during the course. In other words, the degree of inflammation may change with the course of the disease. As in DVT, the IL-6 and CRP levels were higher on admission and then gradually declined during the subsequent days [30].

Moreover, the serum NLR and CSF IgM level were positively correlated with baseline degree of disability on admission. The Hs-CRP level was positively correlated with the development of seizure at baseline. The baseline serum NLR and CSF IgA, CSF IgM, and CSF IgG levels were positively associated with NIHSS. Although the multivariate logistics analysis found no relationship between inflammatory factors and cerebral venous infarction at baseline, the univariate logistics analysis revealed the correlation between cerebral venous infarction at baseline and serum Hs-CRP, CSF IgM, and CSF IgG levels. Hence, it is suggested that a significant correlation between inflammation and severity of CVT may exist. Previous studies have also found the correlation between Hs-CRP level and disability and neurological deficit in cerebrovascular diseases [11, 12]. Currently, Nagai et al. also demonstrated that blood-brain barrier disruption and encephala edema after CVT are linked to the leukocyte–endothelial cell adhesion [31].

Furthermore, the baseline NLR was significantly associated with high risk of poor outcome at discharge. The ROC curve showed that an NLR ≥ 4.205 could better predict poor outcome at discharge, suggesting that baseline inflammation could influence and predict the short-term outcome of CVT. Recently, a retrospective study on the usefulness of NLR in predicting the presence of cerebral venous sinus thrombosis (CVST) found that higher NLRs were significantly and independently related to the presence of CVST, showing that inflammation may play an important role in CVST [8], which was consistent with our findings. Therefore, it is suggested that anti-inflammation probably improves the outcome of CVT in patients without definite inflammatory diseases. Although the use of steroids in CVT is not recommended in the current guideline, it does not mean the denial of the effect of immunoregulation in CVT. This study on steroids aimed at proving its function of decreasing vasogenic edema rather than anti-inflammation [32]. However, the study found no association between baseline inflammation and long-term outcome of these patients.

Our study has some important limitations. First, as a retrospective study, there are some missing data, and we cannot evaluate the outcome at a fixed time point. Second, some patients failed to be followed up due to various reasons. Third, the number of studied inflammatory factors was too small to obtain the ideal conclusion. Lastly, there was not enough study population. However, perhaps the limitations above could partly account for the fact that we did not find a correlation between inflammation and long-term outcome of CVT. Unfortunately, the underlying mechanism of inflammation acting on CVT cannot be precisely explained by this type of clinical study. Therefore, further exploration on this aspect using animal experiments is needed.

## Conclusions

Inflammation may develop soon after the development of CVT and changes with the course of the disease. Moreover, inflammation is correlated with severity and short-term outcome of CVT. Therefore, inflammation may be a promising therapeutic target for CVT in patients without definite inflammatory diseases. However, given the limitations of the study, we need to increase the sample size, explore more inflammatory factors, and conduct a prospective randomized controlled study so that we could draw an expected conclusion.

## Abbreviations

CI: Confidence interval
CSF: Cerebrospinal fluid
Hs-CRP: Hypersensitive C-reactive protein
ICP: Intracranial pressure
Ig: Immunoglobulin
IL-6: Interleukin-6
IQR: Interquartile range
mRS: The modified Rankin Scale
NIHSS: The National Institutes of Health Stroke Score
NLR: The Neutrophil to Lymphocyte ratio
OR: Odds ratio
ROC: Receiver operating characteristic curve

## References

[1] Gustavo Saposnik MD (2011). Diagnosis and management of cerebral venous thrombosis. Stroke.

[2] Suzanne MS, de Sousa DA, José MF (2017). Cerebral venous thrombosis. Neurology.

[3] Chinese society of neurology (2015). The guidance of diagnosis and treatment for cerebral venous thrombosis in China 2015. Chin J Neurol.

[4] Levi M, van der Poll T, Büller HR (2004). Bidirectional relation between inflammation and coagulation. Circulation.

[5] Iadecola C, Anrather J (2011). The immunology of stroke: from mechanisms to translation. Nat Med.

[6] Varma MR, Varga AJ, Knipp BS (2003). Neutropenia impairs venous thrombosis resolution in the rat. J Vasc Surg.

[7] van Aken BE, den Heijer M, Bos GM (2000). Recurrent venous thrombosis and markers of inflammation. Thromb Haemost.

[8] Gupta R, Patadia D, Velayudhan V (2017). Orbital cellulitis, cavernous sinus thrombosis, internal jugular vein thrombus, and clival osteomyelitis secondary to acute sinusitis. Am J Respir Crit Care Med.

[9] Qun S, Tang Y, Sun J (2017). Neutrophil-to-lymphocyte ratio predicts 3-month outcome of acute ischemic stroke. Neurotox Res.

[10] Bisoendial RJ, Kastelein JJ, Levels JH (2005). Activation of inflammation and coagulation after infusion of C-reactive protein in humans. Circ Res.

[11] Pandey A, Shrivastava AK, Saxena K (2014). Neuron specific enolase and C-reactive protein levels in stroke and its subtypes: correlation with degree of disability. Neurochem Res.

[12] Yongjing Z, Wei H (2016). Hs-CRP in stroke: a meta-analysis. Clin Chim Acta.

[13] Van der Poll T, Levi M, Hack CE (1994). Elimination of IL-6 attenuates coagulation in experimental endotoxemia in chimpanzees. J Exp Med.

[14] Stouthard JM, Levi M, Hack CE (1996). Interleukin-6 stimulates coagulation, not fibrinolysis, in humans. Thromb Haemost.

[15] Vormittag R, Hsieh K, Kaider A (2006). Interleukin-6 and interleukin-6 promoter polymorphism (-174) G > C in patients with spontaneous venous thromboembolism. Thromb Haemost.

[16] Çiçek G, Açıkgoz SK, Bozbay M (2015). Neutrophil-lymphocyte ratio and platelet-lymphocyte ratio combination can predict prognosis in patients with ST-segment elevation myocardial infarction undergoing primary percutaneous coronary intervention. Angiology.

[17] Wang F, Hu S, Ding Y (2016). Neutrophil-to-lymphocyte ratio and 30-day mortality in patients with acute intracerebral hemorrhage. J Stroke Cerebrovasc Dis.

[18] Azab B, Shah N, Akerman M (2016). Value of platelet/lymphocyte ratio as a predictor of all-cause mortality after non-ST-elevation myocardial infarction. J Thromb Thrombolysis.

[19] Akboga YE, Bektas H, Anlar O (2017). Usefulness of platelet to lymphocyte and neutrophil to lymphocyte ratios in predicting the presence of cerebral venous sinus thrombosis and in-hospital major adverse cerebral events. J Neurol Sci.

[20] Khasawneh AH, Garling RJ, Harris CA (2018). Cerebrospinal fluid circulation: what do we know and how do we know it?. Brain Circ.

[21] Domenico N (2014). Inflammatory mediators as biomarkers in brain disorders. Inflammation.

[22] Prasad R (1985). Immunoglobulins in certain CNS disorders: a study of CSF Ig classes G, A, M, D, and E concentrations. Am J Clin Pathol.

[23] Alshoabi SA (2017). Cerebral venous sinus thrombosis: a diagnostic challenge in a rare presentation. Brain Circ.

[24] Arauz A (2015). Time to recanalisation in patients with cerebral venous thrombosis under anticoagulation therapy. J Neurol Neurosurg Psychiatr.

[25] Hu Y, Meng R, Zhang X (2017). Serum neuron specific enolase may be a marker to predict the severity and outcome of cerebral venous thrombosis. J Neurol.

[26] Rust Ruslan, Hofer Anna-Sophie, Schwab Martin E. (2018). Stroke Promotes Systemic Endothelial Inflammation and Atherosclerosis. Trends in Molecular Medicine.

[27] Arenillas JF (2015). Intracranial atherosclerosis and inflammation: lessons from the East and the West. Brain Circ.

[28] Khoyetsyan A, Kacimi R, Tsakanova G (2017). Activated complement protein C5a does not affect brain-derived endothelial cell viability and zonula occludens-1 levels following oxygen-glucose deprivation. Brain Circ.

[29] Zhao H, Li G, Ma Q (2017). MicroRNA-99a-5p in circulating immune cells as a potential biomarker for the early diagnosis of ischemic stroke. Brain Circ.

[30] Roumen-Klappe EM, den Heijer M, van Uum SH (2002). Inflammatory response in the acute phase of deep vein thrombosis. J Vasc Surg.

[31] Nagai M, Terao S, Yilmaz G (2010). Roles of inflammation and the activated protein C pathway in the brain edema associated with cerebral venous sinus thrombosis. Stroke.

[32] Canhão P, Cortesão A, Cabral M (2008). Are steroids useful to treat cerebral venous thrombosis?. Stroke.

